# Dibromidobis­(4-hydr­oxy-1,5-dimethyl-2-phenyl-3-pyrazolone)zinc(II)

**DOI:** 10.1107/S1600536808016838

**Published:** 2008-06-07

**Authors:** Pascale Lemoine, Bernard Viossat, Jean Daniel Brion, Alain Bekaert

**Affiliations:** a Laboratoire de Cristallographie et RMN biologiques, UMR 8015 CNRS, Faculté des Sciences Pharmaceutiques et Biologiques de Paris Descartes, 4 avenue de l’Observatoire, 75270 Paris Cedex 06, France; b Université de Paris XI, Faculté des Sciences Pharmaceutiques et Biologiques, Laboratoire de Chimie Thérapeutique BioCIS, UPRES-A 8076 CNRS, 5 rue J. B. Clément, 92296 Châtenay-Malabry Cedex, France

## Abstract

In the title compound, [ZnBr_2_(C_11_H_12_N_2_O_2_)_2_], the Zn(II) ion is coordinated by two Br atoms and two O atoms from two 4-hydroxy­anti­pyrine mol­ecules *via* the carbonyl O atoms, which act as monodentate ligands, giving rise to a distorted tetra­hedral geometry. The values of the bond angles at the Zn atom are in the range 99.4 (1) to 113.2 (1)°. The presence of O—H⋯O and O—H⋯Br intra­molecular hydrogen bonds can explain the difference between the two Zn—O [1.961 (3)/2.015 (3) Å] and the two Zn—Br [2.350 (1)/2.378 (1) Å] bond lengths. The crystal structure is governed by C—H⋯O, C—H⋯Br and Zn—Br⋯*Cg*(π-ring) inter­actions.

## Related literature

For related literature, see: Bekaert *et al.* (2003 ▶, 2007 ▶); Filiz *et al.* (2008 ▶); Lemoine *et al.* (2007 ▶); Matzke *et al.* (2000 ▶); Melov *et al.*, (1998 ▶); Panneerselvam *et al.* (1996 ▶); Tougu *et al.* (2008 ▶).
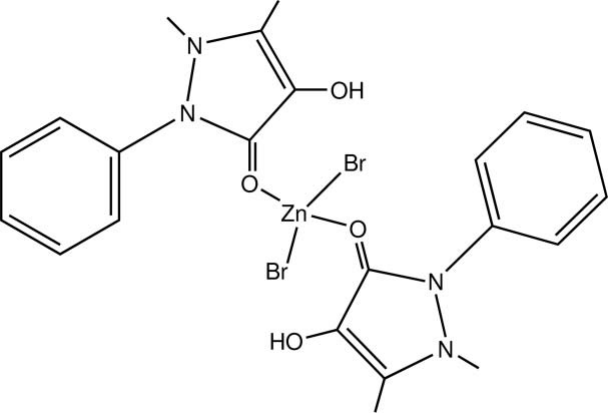


## Experimental

### 

#### Crystal data

[ZnBr_2_(C_11_H_12_N_2_O_2_)_2_]
*M* *_r_* = 633.64
Tetragonal, 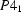

*a* = 9.824 (3) Å
*c* = 26.120 (3) Å
*V* = 2521 (1) Å^3^
*Z* = 4
Mo *K*α radiation
μ = 4.18 mm^−1^
*T* = 293 (2) K
0.17 × 0.16 × 0.15 mm

#### Data collection

Enraf-Nonius CAD-4 diffractometer
Absorption correction: none
15417 measured reflections
7354 independent reflections
3152 reflections with *I* > 2σ(*I*)
*R*_int_ = 0.091
3 standard reflections frequency: 60 min intensity decay: none

#### Refinement

*R*[*F* ^2^ > 2σ(*F* ^2^)] = 0.035
*wR*(*F* ^2^) = 0.093
*S* = 0.90
7354 reflections
304 parameters
1 restraint
H-atom parameters constrained
Δρ_max_ = 0.36 e Å^−3^
Δρ_min_ = −0.30 e Å^−3^
Absolute structure: Flack (1983 ▶), 3602 Friedel pairs
Flack parameter: −0.015 (9)

### 

Data collection: *CAD-4 EXPRESS* (Enraf–Nonius, 1994 ▶); cell refinement: *CAD-4 EXPRESS*; data reduction: *XCAD4* (Harms & Wocadlo, 1995 ▶); program(s) used to solve structure: *SIR92* (Altomare *et al.*, 1994 ▶); program(s) used to refine structure: *SHELXL97* (Sheldrick, 2008 ▶); molecular graphics: *ORTEPIII* (Burnett & Johnson, 1996 ▶) and *ORTEP-3 for Windows* (Farrugia, 1997 ▶); software used to prepare material for publication: *WinGX* (Farrugia, 1999 ▶).

## Supplementary Material

Crystal structure: contains datablocks global, I. DOI: 10.1107/S1600536808016838/dn2351sup1.cif

Structure factors: contains datablocks I. DOI: 10.1107/S1600536808016838/dn2351Isup2.hkl

Additional supplementary materials:  crystallographic information; 3D view; checkCIF report

## Figures and Tables

**Table 1:** Hydrogen-bond geometry (Å, °)

*D*—H⋯*A*	*D*—H	H⋯*A*	*D*⋯*A*	*D*—H⋯*A*
O5—H5⋯O24	0.82	1.94	2.734 (5)	164
O25—H25⋯Br1	0.82	2.40	3.212 (4)	169
C10—H10⋯O5^i^	0.93	2.47	3.378 (8)	165
C27—H27*C*⋯Br2^ii^	0.96	2.81	3.686 (7)	151

Symmetry codes: (i) 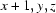; (ii) 
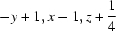
.

**Table 2:** Zn—Br⋯*Cg*(π-ring) interaction

*y*—*X*(*I*)⋯*Cg*(*J*)	*X*⋯*Cg*	*X*-Perp	γ	*Y*—*X*⋯*Cg*	
Zn1—Br1⋯*Cg*1^i^	3.671 (2)	3.646	6.76	132.21 (4)	

Symmetry code: (i) 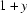, 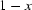, 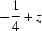. *Cg*1 is the centroid of atoms Cl/N2/N3/C4/C5.

**Fig. 1.**
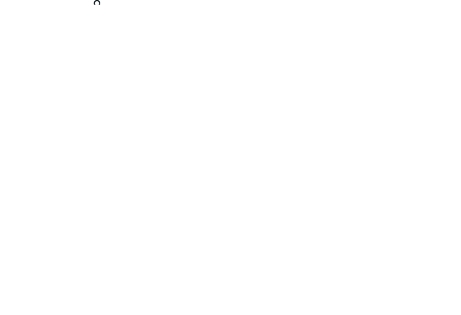
Molecular view of the complex with the atom-labelling scheme. Ellipsoids are drawn at the 50% probability level. Hydrogen bonds are shown as dashed lines. H atoms are represented as small spheres of arbitrary radii.

## supplementary crystallographic information

### Comment

Metals like Zn are expected to be involved in neurodegenerative diseases such as Alzheimer or Parkinson leading to neurofibrillary tangles degeneration and tau protein accumulation (Filiz *et al.*,2008; Tougu *et al.*,2008). These amyloi¨d plaques in the cortical brain are the sign of cerebral aging and associated with a neuronic a high level of metals. Much work (Melov *et al.*, 1998) is now devoted to theses diseases since no real drug is available up to date. As researchers postulate that soft chelating drugs could interfere with free metal accumulation and neuronal collapsus, our idea was that phenazone (antipyrine), a well known antipyretic brain available drug, could become a soft chelating molecule upon hydroxylation in the 4-hydroxy derivative. For this reason and our knowledge in metal amide complexes, (Bekaert *et al.*, 2007; Lemoine *et al.*, 2007) we have prepared a new cristalline complex including Zn and 4-hydroxy-1,5-dimethyl-2-phenyl-3-pyrazolone (4-hydroxyantipyrine) which is of considerable interest as a antipyrine primary metabolite and which is the object of many biological studies the latter years, by example in the evaluation of the influence of diabete mellitus on antipyrine metabolism (Matzke *et al.*, 2000). The hydroxyamide structure which is close to lactamide let us to test it as a metal pinch. Following our work concerning lactamide and zinc(II) complex (Bekaert *et al.*, 2003), we now report a new zinc complex with 4-hydroxyantipyrine.

The title compound contains one monomeric tetrahedral zinc complex, [Zn(C_22_H_24_N_4_O_4_)Br_2_]. The Zn atom is surrounded by two monodentate 4-hydroxyantipyrine ligands *via* the carbonyl O atom O4 (or O24) in the *sp*^2^ lone-pair direction and two Br ligands (Fig. 1). The complex exhibits a distorted tetrahedral geometry around the zincII atom. The degree of deviation from an ideal tetrahedron is appreciable with the angles around Zn atom ranging from 99.4 (1) to 113.2 (1) °. The Zn—O and Zn—Br distances in the coordination polyhedron are 1.961 (3)/2.015 (3) Å and 2.351 (1)/2.379 (1)Å, respectively, in good agreement with those found in similar Zn^II^tetrahedral coordination (Bekaert *et al.*, 2003). The difference between the two Zn—O (or the two Zn—Br) bond lengths can be explained by the presence of the O5—H5···O24 (or O25—H25···Br1) intramolecular hydrogen bond (Table 1) which causes the stretching of the Zn—O24 (or Zn—Br1) bond. Each hydroxyantipyrine ligand consists of a pyrazole P1 (Cl/N2—N3/C4—C5) [or P3 (C2l/N22—N23/C24—C25)] and a phenyl ring P2 (C8—C13) [or P4 (C28—C33)] which are planar with maximum deviation of 0.017 (3) Å for N2 (first ligand) and 0.021 (3) Å for N23 (second ligand). The dihedral angles are 65.2 (2)° between P1 and P2 and 81.6 (2)° for P3 and P4, these values are significantly different from those reported in 4-hydroxyantipyrine [42.5 (1)°] (Panneerselvam *et al.*,1996).

The crystal packing is governed by weak C—H···O and Zn—Br···*Cg1*( centroid of the P1 plane) interactions (Tables1 and 2).

### Experimental

The title compound, dibromido-bis[4-hydroxyantipyrine]zinc(II), was prepared by mixing 1.02 g (5 mmole) of 4-hydroxyantipyrine dissolved in hot acetic acid (10 ml, 353 K) and 10 ml of a solution of ZnBr2 (0.496 g, 2 mmole) in boiling acetic acid. Upon slow cooling, crystal suitable for X-ray diffraction were recovered.

### Refinement

All H atoms were positioned geometrically and treated as riding on their parent atoms with distances C—H = 0.96 Å (CH_3_) and *U*_iso_(H) = 1.5 times *U*_eq_(C) or 0.93 Å (aromatic) with *U*_iso_(H) = 1.2 times *U*_eq_(C) and O—H= 0.82Å with *U*_iso_(H) = 1.5 times *U*_eq_(O).

### Crystal data

**Table:**

[ZnBr_2_(C_11_H_12_N_2_O_2_)_2_]	*Z* = 4
*M**_r_* = 633.64	*F*_000_ = 1264
Tetragonal, *P*4_1_	*D*_x_ = 1.670 Mg m^−^^3^
Hall symbol: P4w	Mo *K*α radiation λ = 0.71073 Å
*a* = 9.824 (3) Å	Cell parameters from 25 reflections
*b* = 9.824 (3) Å	θ = 2.2–7.0º
*c* = 26.120 (3) Å	µ = 4.18 mm^−^^1^
α = 90º	*T* = 293 (2) K
β = 90º	Parallelepiped, colourless
γ = 90º	0.18 × 0.16 × 0.15 mm
*V* = 2521 (1) Å^3^	

### Data collection

**Table:**

Enraf-Nonius CAD-4 diffractometer	*R*_int_ = 0.091
Radiation source: fine-focus sealed tube	θ_max_ = 30.1º
Monochromator: graphite	θ_min_ = 2.2º
*T* = 293(2) K	*h* = −13→13
ω – 2θ scans	*k* = 0→13
Absorption correction: none	*l* = −36→36
15417 measured reflections	3 standard reflections
7354 independent reflections	every 60 min
3152 reflections with *I* > 2σ(*I*)	intensity decay: none

### Refinement

**Table:**

Refinement on *F*^2^	Hydrogen site location: inferred from neighbouring sites
Least-squares matrix: full	H-atom parameters constrained
*R*[*F*^2^ > 2σ(*F*^2^)] = 0.035	*w* = 1/[σ^2^(*F*_o_^2^) + (0.0396*P*)^2^] where *P* = (*F*_o_^2^ + 2*F*_c_^2^)/3
*wR*(*F*^2^) = 0.093	(Δ/σ)_max_ = 0.023
*S* = 0.90	Δρ_max_ = 0.36 e Å^−^^3^
7354 reflections	Δρ_min_ = −0.30 e Å^−^^3^
304 parameters	Extinction correction: none
1 restraint	Absolute structure: Flack (1983), 3602 Friedel pairs
Primary atom site location: structure-invariant direct methods	Flack parameter: −0.015 (9)
Secondary atom site location: difference Fourier map	

### Special details

**Table:**

Geometry. All esds (except the esd in the dihedral angle between two l.s. planes) are estimated using the full covariance matrix. The cell esds are taken into account individually in the estimation of esds in distances, angles and torsion angles; correlations between esds in cell parameters are only used when they are defined by crystal symmetry. An approximate (isotropic) treatment of cell esds is used for estimating esds involving l.s. planes.
Refinement. Refinement of *F*^2^ against ALL reflections. The weighted *R*-factor *wR* and goodness of fit *S* are based on *F*^2^, conventional *R*-factors *R* are based on *F*, with *F* set to zero for negative *F*^2^. The threshold expression of *F*^2^ > σ(*F*^2^) is used only for calculating *R*-factors(gt) *etc*. and is not relevant to the choice of reflections for refinement. *R*-factors based on *F*^2^ are statistically about twice as large as those based on *F*, and *R*- factors based on ALL data will be even larger.

### Fractional atomic coordinates and isotropic or equivalent isotropic displacement parameters (Å^2^)

**Table:**

	*x*	*y*	*z*	*U*_iso_*/*U*_eq_	
Zn1	0.74076 (5)	0.20414 (5)	0.423054 (19)	0.05571 (13)	
Br1	0.88194 (6)	0.16046 (6)	0.35092 (2)	0.07972 (17)	
Br2	0.57242 (7)	0.36720 (6)	0.40493 (2)	0.0908 (2)	
C1	0.6782 (5)	0.2246 (5)	0.6042 (2)	0.0645 (13)	
N2	0.8063 (4)	0.2622 (5)	0.61659 (16)	0.0699 (11)	
N3	0.8827 (4)	0.2682 (4)	0.57175 (14)	0.0579 (9)	
O4	0.8439 (3)	0.2507 (3)	0.48491 (13)	0.0627 (8)	
C4	0.7980 (5)	0.2417 (4)	0.53136 (18)	0.0531 (11)	
O5	0.5526 (3)	0.1862 (4)	0.52624 (16)	0.0774 (10)	
H5	0.5683	0.1309	0.5035	0.116*	
C5	0.6701 (4)	0.2140 (4)	0.5522 (2)	0.0574 (12)	
C6	0.5746 (6)	0.1951 (7)	0.6436 (2)	0.097 (2)	
H6A	0.5993	0.1139	0.6618	0.146*	
H6B	0.4877	0.1824	0.6275	0.146*	
H6C	0.5694	0.2700	0.6672	0.146*	
C7	0.8723 (6)	0.2520 (7)	0.6664 (2)	0.0960 (18)	
H7A	0.8082	0.2746	0.6928	0.144*	
H7B	0.9477	0.3140	0.6677	0.144*	
H7C	0.9045	0.1607	0.6714	0.144*	
C8	1.0114 (4)	0.3326 (5)	0.57024 (18)	0.0565 (11)	
C9	1.1196 (6)	0.2603 (7)	0.5512 (2)	0.094 (2)	
H9	1.1110	0.1706	0.5403	0.113*	
C10	1.2471 (7)	0.3318 (13)	0.5490 (3)	0.135 (4)	
H10	1.3244	0.2882	0.5367	0.162*	
C11	1.2545 (10)	0.4602 (14)	0.5647 (3)	0.140 (4)	
H11	1.3380	0.5045	0.5626	0.168*	
C12	1.1441 (9)	0.5314 (8)	0.5840 (3)	0.111 (3)	
H12	1.1530	0.6205	0.5956	0.133*	
C13	1.0223 (6)	0.4661 (6)	0.5853 (2)	0.0779 (15)	
H13	0.9453	0.5123	0.5966	0.093*	
C21	0.8032 (5)	−0.2887 (4)	0.4583 (2)	0.0630 (12)	
N22	0.6796 (4)	−0.3069 (4)	0.47977 (18)	0.0638 (11)	
N23	0.6143 (4)	−0.1837 (4)	0.47984 (16)	0.0595 (10)	
O24	0.6506 (3)	0.0316 (3)	0.44738 (13)	0.0597 (8)	
C24	0.6943 (4)	−0.0888 (4)	0.45531 (17)	0.0487 (10)	
O25	0.9281 (3)	−0.1073 (3)	0.41933 (19)	0.0825 (10)	
H25	0.9098	−0.0349	0.4053	0.124*	
C25	0.8144 (4)	−0.1576 (5)	0.4424 (2)	0.0602 (12)	
C26	0.9040 (6)	−0.4025 (5)	0.4535 (3)	0.099 (2)	
H26A	0.9660	−0.3830	0.4260	0.148*	
H26B	0.8568	−0.4859	0.4465	0.148*	
H26C	0.9539	−0.4112	0.4849	0.148*	
C27	0.6348 (7)	−0.4141 (5)	0.5139 (3)	0.0872 (18)	
H27A	0.6787	−0.4979	0.5047	0.131*	
H27B	0.5380	−0.4247	0.5111	0.131*	
H27C	0.6581	−0.3907	0.5485	0.131*	
C28	0.4741 (5)	−0.1712 (4)	0.49287 (19)	0.0572 (12)	
C29	0.4387 (6)	−0.1184 (6)	0.5390 (3)	0.0860 (18)	
H29	0.5048	−0.0915	0.5624	0.103*	
C30	0.3006 (9)	−0.1055 (7)	0.5503 (3)	0.110 (3)	
H30	0.2737	−0.0673	0.5813	0.132*	
C31	0.2057 (7)	−0.1483 (7)	0.5167 (4)	0.107 (3)	
H31	0.1142	−0.1402	0.5253	0.128*	
C32	0.2391 (6)	−0.2030 (8)	0.4702 (3)	0.108 (2)	
H32	0.1727	−0.2318	0.4472	0.129*	
C33	0.3770 (6)	−0.2138 (6)	0.4589 (3)	0.0827 (16)	
H33	0.4037	−0.2506	0.4277	0.099*	

### Atomic displacement parameters (Å^2^)

**Table:**

	*U*^11^	*U*^22^	*U*^33^	*U*^12^	*U*^13^	*U*^23^
Zn1	0.0566 (3)	0.0519 (3)	0.0587 (3)	−0.0067 (2)	−0.0048 (2)	0.0016 (2)
Br1	0.0845 (4)	0.0840 (4)	0.0706 (4)	0.0026 (3)	0.0170 (3)	0.0095 (3)
Br2	0.0966 (4)	0.0866 (4)	0.0893 (5)	0.0281 (3)	−0.0168 (3)	−0.0011 (3)
C1	0.064 (3)	0.062 (3)	0.068 (4)	−0.011 (2)	0.008 (3)	−0.009 (2)
N2	0.077 (3)	0.077 (3)	0.056 (3)	−0.012 (2)	0.012 (2)	−0.005 (2)
N3	0.053 (2)	0.073 (2)	0.048 (3)	−0.0083 (17)	0.0001 (18)	0.0005 (18)
O4	0.0571 (18)	0.073 (2)	0.058 (2)	−0.0122 (15)	0.0017 (15)	−0.0002 (16)
C4	0.063 (3)	0.043 (2)	0.053 (3)	0.0022 (19)	0.001 (2)	0.000 (2)
O5	0.0492 (19)	0.093 (3)	0.090 (3)	−0.0133 (17)	0.0043 (18)	−0.013 (2)
C5	0.041 (3)	0.051 (3)	0.080 (4)	−0.0038 (19)	0.008 (2)	−0.001 (2)
C6	0.088 (4)	0.109 (4)	0.095 (5)	−0.034 (3)	0.034 (4)	−0.013 (4)
C7	0.101 (4)	0.138 (5)	0.049 (4)	−0.019 (4)	0.007 (3)	0.002 (3)
C8	0.055 (3)	0.062 (3)	0.052 (3)	−0.002 (2)	−0.001 (2)	0.001 (2)
C9	0.067 (4)	0.136 (6)	0.078 (4)	0.027 (4)	−0.004 (3)	−0.034 (4)
C10	0.058 (4)	0.255 (11)	0.092 (6)	0.006 (5)	0.010 (3)	−0.048 (7)
C11	0.113 (7)	0.249 (12)	0.057 (5)	−0.082 (8)	0.006 (4)	0.001 (6)
C12	0.142 (7)	0.107 (5)	0.084 (5)	−0.064 (5)	0.000 (5)	0.000 (4)
C13	0.091 (4)	0.072 (3)	0.071 (4)	−0.023 (3)	−0.002 (3)	0.001 (3)
C21	0.055 (3)	0.050 (3)	0.084 (4)	0.003 (2)	−0.003 (3)	0.003 (2)
N22	0.064 (3)	0.041 (2)	0.086 (3)	0.0001 (18)	0.010 (2)	0.0034 (19)
N23	0.062 (2)	0.044 (2)	0.072 (3)	−0.0030 (17)	0.0102 (19)	−0.0017 (18)
O24	0.0507 (16)	0.0470 (16)	0.081 (2)	−0.0025 (13)	0.0073 (15)	−0.0007 (15)
C24	0.048 (2)	0.044 (2)	0.053 (3)	−0.0052 (19)	−0.0045 (19)	−0.0060 (19)
O25	0.0525 (17)	0.073 (2)	0.122 (3)	0.0007 (15)	0.021 (2)	0.020 (2)
C25	0.052 (3)	0.059 (3)	0.069 (3)	−0.001 (2)	−0.001 (2)	0.001 (2)
C26	0.086 (4)	0.059 (3)	0.150 (7)	0.019 (3)	0.024 (4)	0.020 (4)
C27	0.110 (5)	0.055 (3)	0.097 (5)	0.001 (3)	0.017 (4)	0.009 (3)
C28	0.058 (3)	0.048 (2)	0.066 (3)	−0.006 (2)	0.014 (2)	0.005 (2)
C29	0.086 (4)	0.083 (4)	0.089 (5)	−0.010 (3)	0.024 (3)	−0.017 (3)
C30	0.119 (6)	0.075 (4)	0.136 (7)	0.006 (4)	0.066 (5)	−0.007 (4)
C31	0.068 (4)	0.083 (4)	0.170 (9)	0.002 (3)	0.037 (5)	0.023 (5)
C32	0.069 (4)	0.129 (6)	0.125 (7)	−0.021 (4)	−0.006 (4)	0.044 (5)
C33	0.068 (3)	0.093 (4)	0.088 (5)	−0.017 (3)	0.011 (3)	0.002 (3)

### Geometric parameters (Å, °)

**Table:**

Zn1—O4	1.961 (3)	C12—H12	0.9300
Zn1—O24	2.015 (3)	C13—H13	0.9300
Zn1—Br2	2.3505 (10)	C21—N22	1.349 (6)
Zn1—Br1	2.3786 (8)	C21—C25	1.357 (7)
C1—N2	1.350 (6)	C21—C26	1.498 (7)
C1—C5	1.365 (7)	N22—N23	1.370 (5)
C1—C6	1.477 (7)	N22—C27	1.448 (7)
N2—N3	1.392 (5)	N23—C24	1.378 (5)
N2—C7	1.457 (7)	N23—C28	1.424 (6)
N3—C4	1.369 (6)	O24—C24	1.275 (5)
N3—C8	1.415 (6)	C24—C25	1.400 (6)
O4—C4	1.297 (5)	O25—C25	1.363 (6)
C4—C5	1.396 (7)	O25—H25	0.8200
O5—C5	1.366 (6)	C26—H26A	0.9600
O5—H5	0.8200	C26—H26B	0.9600
C6—H6A	0.9600	C26—H26C	0.9600
C6—H6B	0.9600	C27—H27A	0.9600
C6—H6C	0.9600	C27—H27B	0.9600
C7—H7A	0.9600	C27—H27C	0.9600
C7—H7B	0.9600	C28—C29	1.356 (7)
C7—H7C	0.9600	C28—C33	1.368 (8)
C8—C9	1.372 (7)	C29—C30	1.395 (9)
C8—C13	1.373 (7)	C29—H29	0.9300
C9—C10	1.437 (11)	C30—C31	1.349 (12)
C9—H9	0.9300	C30—H30	0.9300
C10—C11	1.328 (14)	C31—C32	1.368 (11)
C10—H10	0.9300	C31—H31	0.9300
C11—C12	1.386 (13)	C32—C33	1.390 (9)
C11—H11	0.9300	C32—H32	0.9300
C12—C13	1.358 (9)	C33—H33	0.9300
			
O4—Zn1—O24	99.41 (13)	C12—C13—C8	120.9 (6)
O4—Zn1—Br2	111.74 (10)	C12—C13—H13	119.6
O24—Zn1—Br2	109.12 (8)	C8—C13—H13	119.6
O4—Zn1—Br1	113.16 (10)	N22—C21—C25	109.0 (4)
O24—Zn1—Br1	110.75 (9)	N22—C21—C26	122.0 (4)
Br2—Zn1—Br1	111.94 (3)	C25—C21—C26	129.0 (5)
N2—C1—C5	108.3 (4)	C21—N22—N23	107.8 (3)
N2—C1—C6	121.9 (5)	C21—N22—C27	128.8 (4)
C5—C1—C6	129.7 (5)	N23—N22—C27	119.9 (4)
C1—N2—N3	108.2 (4)	N22—N23—C24	109.2 (3)
C1—N2—C7	127.6 (4)	N22—N23—C28	122.0 (4)
N3—N2—C7	120.9 (4)	C24—N23—C28	127.2 (4)
C4—N3—N2	108.2 (4)	C24—O24—Zn1	133.1 (3)
C4—N3—C8	127.4 (4)	O24—C24—N23	120.7 (4)
N2—N3—C8	121.6 (4)	O24—C24—C25	133.8 (4)
C4—O4—Zn1	125.1 (3)	N23—C24—C25	105.4 (4)
O4—C4—N3	119.8 (4)	C25—O25—H25	109.5
O4—C4—C5	133.7 (4)	C21—C25—O25	123.1 (4)
N3—C4—C5	106.5 (4)	C21—C25—C24	108.5 (4)
C5—O5—H5	109.5	O25—C25—C24	128.4 (4)
C1—C5—O5	123.9 (4)	C21—C26—H26A	109.5
C1—C5—C4	108.7 (4)	C21—C26—H26B	109.5
O5—C5—C4	127.3 (5)	H26A—C26—H26B	109.5
C1—C6—H6A	109.5	C21—C26—H26C	109.5
C1—C6—H6B	109.5	H26A—C26—H26C	109.5
H6A—C6—H6B	109.5	H26B—C26—H26C	109.5
C1—C6—H6C	109.5	N22—C27—H27A	109.5
H6A—C6—H6C	109.5	N22—C27—H27B	109.5
H6B—C6—H6C	109.5	H27A—C27—H27B	109.5
N2—C7—H7A	109.5	N22—C27—H27C	109.5
N2—C7—H7B	109.5	H27A—C27—H27C	109.5
H7A—C7—H7B	109.5	H27B—C27—H27C	109.5
N2—C7—H7C	109.5	C29—C28—C33	120.9 (5)
H7A—C7—H7C	109.5	C29—C28—N23	119.6 (5)
H7B—C7—H7C	109.5	C33—C28—N23	119.5 (4)
C9—C8—C13	122.5 (5)	C28—C29—C30	118.2 (7)
C9—C8—N3	118.1 (5)	C28—C29—H29	120.9
C13—C8—N3	119.2 (4)	C30—C29—H29	120.9
C8—C9—C10	115.8 (7)	C31—C30—C29	120.4 (7)
C8—C9—H9	122.1	C31—C30—H30	119.8
C10—C9—H9	122.1	C29—C30—H30	119.8
C11—C10—C9	120.0 (7)	C30—C31—C32	122.4 (6)
C11—C10—H10	120.0	C30—C31—H31	118.8
C9—C10—H10	120.0	C32—C31—H31	118.8
C10—C11—C12	123.3 (7)	C31—C32—C33	116.9 (7)
C10—C11—H11	118.4	C31—C32—H32	121.6
C12—C11—H11	118.4	C33—C32—H32	121.6
C13—C12—C11	117.4 (7)	C28—C33—C32	121.2 (7)
C13—C12—H12	121.3	C28—C33—H33	119.4
C11—C12—H12	121.3	C32—C33—H33	119.4

### Hydrogen-bond geometry (Å, °)

**Table:**

*D*—H···*A*	*D*—H	H···*A*	*D*···*A*	*D*—H···*A*
O5—H5···O24	0.82	1.94	2.734 (5)	164
O25—H25···Br1	0.82	2.40	3.212 (4)	169
C10—H10···O5^i^	0.93	2.47	3.378 (8)	165
C27—H27C···Br2^ii^	0.96	2.81	3.686 (7)	151

Symmetry codes: (i) *x*+1, *y*, *z*; (ii) −*y*+1, *x*−1, *z*+1/4.

### Table 2 Zn—Br···Cg(π-ring) interaction

**Table:**

Y—X(I)···Cg(J)	X···Cg	X-Perp	Gamma	Y—X···Cg	
Zn1—Br1···Cg1^i^	3.671 (2)	3.646	6.76	132.21 (4)	

Symmetry code: (i) 1+y, 1-x, -1/4 + z. Cg1 is the centroid of atoms Cl/N2/N3/C4/C5.
